# Hospital Investment Decisions and Prioritization of Clinical Programs

**DOI:** 10.7759/cureus.79998

**Published:** 2025-03-03

**Authors:** Bishan Nandy

**Affiliations:** 1 Hospital Administration, University of Illinois Hospital & Health Sciences System, Chicago, USA

**Keywords:** clinical program prioritization, decision-making frameworks, evidence-based frameworks, financial constraints, healthcare 4.0 technologies, healthcare sustainability, health technology assessment, hospital investment, predictive analytics, resource allocation

## Abstract

Making investment decisions in hospitals and prioritizing clinical programs are crucial to ensure optimal healthcare delivery, responsible use of resources, and long-term sustainability of an organization. This review examines the existing literature to identify the key factors influencing investment decisions, evaluates evidence-based frameworks for prioritizing resources, and articulates challenges and future directions for healthcare organizations. Critical drivers of hospital investments include financial health, demographics, ownership structure, and technology adoption. However, program budgeting and marginal analysis, health technology assessment, multi-criteria decision analysis, and evidence-based design frameworks will ensure that decisions are systematic and transparent. These tools help hospitals balance infrastructure investments, technology, specialized clinical programs, and emergency response. The dependence on past allocation practices, funding constraints, and stakeholder misalignment inhibits the best decision-making. For those barriers to be addressed, predictive analytics and artificial intelligence must already be in the evaluation process, as should configuration processes for interrelated tech, and collaboration among stakeholders should be fostered. Furthermore, investments aligned with sustainability principles and equity goals will be more resilient and adaptive to changes in the healthcare landscape. This review highlights the importance of healthcare organizations implementing holistic, evidence-based frameworks to guide investment decisions, including low-hanging fruit from today and industry best practices for tomorrow, shifting a provider’s focus to ensure optimal patient outcomes, operational efficiencies, and sustainable growth.

## Introduction and background

Investment decisions in hospitals have a direct and lasting impact on patient care, hospital sustainability, and healthcare innovation. These decisions determine how resources are allocated, shape the adoption of new medical technologies, and influence the expansion and development of critical clinical services. As healthcare demands increase and financial pressures mount, hospitals must adopt structured and data-driven approaches to investment and resource allocation.

Hospitals operate in a complex environment where financial constraints, technological advancements, and changing patient demographics create competing priorities. Poor investment choices can lead to inefficiencies, wasted resources, and disparities in access to care, while strategic investments can enhance patient outcomes, improve operational efficiency, and drive long-term sustainability [1].

For example, hospitals using program budgeting and marginal analysis (PBMA) to evaluate oncology services have successfully reallocated funds to expand high-impact cancer treatments while cutting costs in underutilized areas [2]. This evidence-based approach ensures that limited healthcare funds are directed toward interventions that provide the greatest benefit.

With limited resources, hospitals must prioritize clinical programs that offer the most significant impact. Health technology assessment (HTA) and PBMA provide structured approaches to decision-making, helping hospitals assess costs, benefits, and long-term value [3]. However, many hospitals still rely on outdated allocation models, historical spending patterns, and ad hoc decision-making, which may not align with patient needs or healthcare advancements [4].

To improve decision-making, value-based frameworks like evidence-based design (EBD) enable hospitals to evaluate both clinical and economic outcomes. Investments in infection-resistant hospital design, for example, have been shown to reduce hospital-acquired infections (HAIs), enhance patient safety, and lower long-term costs [5].

This review examines the key factors influencing hospital investment decisions, evaluates evidence-based prioritization frameworks, and discusses challenges and future directions in resource allocation. It aims to (1) identify financial, market, technological, and organizational factors affecting investment decisions; (2) explore PBMA, HTA, MCDA, and EBD as decision-making tools; (3) highlight barriers to effective investment strategies and propose solutions using predictive analytics and AI-driven frameworks; and (4) demonstrate how integrating financial and operational considerations with evidence-based decision-making enables hospitals to navigate resource constraints, enhance efficiency, and improve patient care outcomes.

## Review

Factors that affect the decision-making process in hospitals

Investment decisions in hospitals are influenced by multiple financial, market, technological, and organizational factors, each playing a critical role in determining how resources are allocated for infrastructure, clinical programs, and technological adoption. Understanding these factors is essential for ensuring strategic alignment with long-term institutional goals, sustainability, and patient care needs [1,4].

Financial Health and Liquidity

Financial stability is a key determinant in hospital investment decisions. Liquidity and cash flow influence whether a hospital can allocate capital to infrastructure improvements, new clinical programs, and advanced medical technologies [6]. The relationship between financial health and liquidity in capital investments has been well established in previous studies [7,8], where financially stronger hospitals with more liquidity invest in capital projects to enhance service delivery and operational capacity.

However, small, independent, and rural hospitals face significant financial constraints, limiting their ability to fund necessary improvements [7]. These hospitals often experience higher agency costs and reduced financing options, which make them vulnerable to market volatility and operational pressures. Capital investment decisions in such settings require careful trade-offs between short-term operational costs and long-term financial sustainability [9]. High-return investments, such as technological upgrades and infrastructure expansion, may be deprioritized due to immediate budgetary constraints [10,11].

Li (2023) discusses capital budgeting, sensitivity analysis, and financial risk assessments in hospital investment decisions [12]. This reference has been added to the section on cost-benefit and risk assessment models to provide a more comprehensive financial evaluation framework.

Market and Demographic Influences

Market dynamics and demographic changes significantly influence hospital investment decisions. One of the most notable demographic trends is the increase in aging populations, particularly individuals aged 65 and older [13]. This trend is leading to increasing demand for medical services, especially chronic disease management, elderly care, and long-term care facilities [8,9]. In response to these transitions, hospitals shift their investments to clinical programs and infrastructure that cater to the needs of older individuals.

Moreover, the supply of primary care physicians and the parallels and shifts in primary care delivery affect investments in hospitals. More primary care providers or increased access to them means the burden on hospitals would decrease, and they would be able to concentrate on the specialized services they offer. In contrast, physician shortage areas and areas with limited access to primary care experience more stress in their hospital systems, requiring investments in programs designed to fill the gap in service [8]. Increases in population size and growth exacerbate these pressures, drivers of expansion in hospitals and their capacity and services needed to meet the growing demand for health services.

Technological Factors

In parallel, we will see the emergence of Healthcare 4.0 (H4.0) technologies, which have brought new opportunities and challenges to hospital investment decision-making [14]. H4. 0 includes innovative technologies like electronic health records (EHRs), telemedicine, and artificial intelligence (AI), as well as digital platforms for managing healthcare data [14]. While these advancements hold great potential to improve patient care, optimize operations, and cut costs, many hospitals face challenges in assessing the technologies’ long-term value and ability to integrate them into existing workflows [15,16].

The main complication has to do with uncertainty about technological investments and asset specificities. Industry-level specificity or investment risk (high asset specificity), that is, investments are made for a very specific function/service, which reduces flexibility and increases the risk of technological adoption [17]. Health systems with less negotiating leverage may prefer outsourcing models so they do not face high upfront costs. Alternatively, most decision-making frameworks do not consider the link between technologies from a value-creation perspective and simply perform cost analyses in isolation [14]. Hospitals require holistic methodologies to tackle these challenges, which include technological interdependencies, uncertainties, and stakeholders' participation.

Kuotu and Lower (2020) examine the return on investment (ROI) from clinical decision-support technologies, helping hospitals evaluate cost-effectiveness and long-term financial returns [18]. This study has been integrated into the HTA section to highlight financial considerations in healthcare IT investments.

Hospital Ownership and Reimbursement Systems

The structure of ownership and reimbursement models are very important factors that influence hospital investment priorities [19]. Between financial motivations and operational goals, for-profit and not-for-profit hospitals share different goals, which lead to different decision strategies. Profit-oriented hospitals are more influenced by the generosity of reimbursement models and cost-based payment schemes because of their profitability motives [20]. These hospitals are more likely to invest in programs and technologies that have a clear financial ROI or improve their market competitiveness.

Nonprofit hospitals, by contrast, prioritize mission-driven investments, putting community long-term benefits ahead of immediate financial returns [21,22]. Such hospitals are also more likely to allocate resources to support under-prioritized populations, such as mental ill health, primary care services, and preventative care programs [4]. The reimbursement model (i.e., cost based or charge based) also plays a vital role in investment decisions [19]. For-profit hospitals are incentivized to invest in systems that generate profits, and not-for-profits are held accountable for both revenue and community objectives.

Marques et al. (2021) explore investment decision-making in private hospitals, providing valuable insights into financial strategies [23].

Alignment of strategy with the organization

Hospitals should have investment decisions that fit a more significant strategic and organizational mission. How patients are prioritized is shaped by the socioeconomic and cultural backdrop where hospitals function and the institutional mission they serve [4]. Patients and employees create motivators for hospitals to allocate their investments wisely, maintaining a balance between short- and long-term sustainability of the hospital with the ability to optimize patient outcomes, operational efficiency, and institutional growth [24].

Strategic Alignment Through Evidence-Based Frameworks

Evidence-based strategic alignment ensures that hospital investments align with organizational priorities to expand service capabilities, increase patient safety, and ensure regulatory compliance [25]. For example, evidence-based frameworks such as HTA allow hospitals to align investments with clinically and economically feasible outcomes [1]. For example, prioritization frameworks such as PBMA enable hospitals to hone in on high-value programs that meet organizational goals while identifying targets for disinvestment from domains of low or poor performance [2].

Real-world example: strategic investment in oncology services: A leading hospital implemented PBMA to optimize oncology services while reallocating funds from underutilized departments. By analyzing marginal benefits, the hospital redirected resources from low-impact surgical expansions toward specialized cancer treatment programs, resulting in (1) increased early cancer detection rates; (2) improved patient survival outcomes; and (3) better cost efficiency in resource utilization.

This case highlights how evidence-based strategic planning can enhance clinical effectiveness while maintaining financial discipline.

Incorporating community and socioeconomic needs: Investment decisions must also consider community healthcare gaps, ensuring equitable access to essential services. Hospitals in underserved regions must prioritize investments in primary care, mental health services, and telemedicine to bridge access disparities [4].

For example, hospitals in rural areas investing in telehealth platforms have seen reduced patient travel time, enhanced specialist access, and improved chronic disease management [26].

When deciding where to invest, facilities must also consider the socioeconomic and cultural requirements of their communities. This entails identifying and closing gaps in healthcare access, expanding investment to underserved services, and leveraging technologies that span the care continuum for diverse patient populations [4]. Investing in areas that matter to the organization and context by aligning investment strategies with organizational and contextual priorities, hospitals can improve their overall performance and build stronger relationships with their communities [26].

Frameworks for prioritizing clinical programs: Decision-making on investment in healthcare providers is a complex process that entails structured frameworks to inform resource allocation, ensuring value optimization. Methods like EBD, PBMA, HTA, multi-criteria decision analysis (MCDA), and risk assessment frameworks are critical to focusing investments in alignment with institutional goals and improving patient outcomes through best practices [3,3,27]. Enabling informed decision-making from a financial, operational, and ethical perspective, these tools empower health system organizations with an ability to consider competing factors to be weighed against one another [1,2,5].

EBD: EBD incorporates scientific evidence and research into investments in healthcare facilities and programs. It is called EBD, which aims to design healthcare environments that improve safety, quality, and operating efficiency while minimizing costs and enhancing patient outcomes [5]. Examples include hospitals that apply EBD concepts and place the highest value on interventions that reduce HAIs, increase patient safety, and decrease the number of falls. These advantages are backed by data and measurable, impact-driven outcomes and enable hospitals to provide stakeholders with long-term value [28,29].

For instance, hospitals implementing infection-resistant materials and improved lighting designs have reported reductions in HAIs and enhanced patient recovery times.

EBD also promotes life-cycle cost analysis, which considers both the initial and long-term operational savings of infrastructure and program investments. For example, hospitals investing in antimicrobial flooring have observed reduced transmission of pathogens, leading to lower infection rates and operational savings [5]. Incorporating robust evidence into decision-making mechanisms can help hospitals make a stronger business case for specific investments by highlighting potential improvements in clinical outcomes and financial performance [30]. An example of this would be the initial expense of using materials that are resistant to infection in designing a hospital, which can lead to long-term cost savings through their impact on healthcare-associated infections and patient recovery time [5].

PBMA: PBMA is a resource allocation framework that evaluates healthcare programs based on their costs and benefits. Through the use of PBMA, healthcare organizations can identify programs with high impact and prioritize investments while also assessing opportunities for the disinvestment of low-value services [2,31]. This approach is especially important in resource-constrained settings, where even small effects can be meaningful with limited budgets.

PBMA is a systematic approach for appraising the marginal benefits derived from reallocating resources across programs. For example, hospitals implementing PBMA in oncology services have successfully shifted resources from low-impact diagnostic services to advanced targeted therapies, improving patient outcomes [1]. For example, funds could get diverted away from initiatives that have been shown to have a limited impact and shift toward ones that have been proven to greatly improve patient outcomes or operational efficiency [1].

The framework also facilitates evidence-informed decision-making by integrating clinical and economic data into the prioritization process. For example, PBMA may help hospitals determine whether funding a new mental health program will have a greater impact than continuing funding for a duplicate service. Those analyses would make sure that expenditures on healthcare investment are in line with the strategic objectives and fill gaps in the delivery of care.

Despite its benefits, PBMA is not utilized frequently in many healthcare systems, which continue to use historical allocation patterns and ad hoc decision-making processes [1]. Although the PBMA framework may seem impractical, the increasing demand for transparent and organized approaches to decision-making makes it a powerful tool to maximize resources available to health care and ensure fair allocation of those resources [32].

HTA: HTA is an integrated process for assessing the clinical, economic, and ethical aspects of healthcare technologies and interventions. HTA bridges the gap of scientific proof and thus allows the health systems to direct investments toward interventions that result in top-tier outcomes, cost-effectiveness, and sustainability [1,33].

HTA is a systematic evaluation process used to assess the benefits, costs, and impact of new technologies (such as medical devices, telemedicine platforms, and digital health tools) in terms of their clinical effectiveness in multiple settings over time. For example, HTA by hospitals may guide the decision to invest in EHRs, weighing the estimated initial cost against anticipated enhancements in patient safety and care coordination [14].

However, resource-limited hospitals struggle with HTA adoption due to high evaluation costs and limited technical expertise [2].

Moreover, HTA increases transparency and accountability through its multi-stakeholder involvement (including clinicians, administrators, policymakers, etc.) in the evaluation process [2].

Although HTA has been widely adopted in high-income countries, it has been adopted rather inconsistently in resource-limited settings. The expanded application of HTA frameworks is vital to both optimizing investment in novel technologies and ensuring the availability of healthcare that is equitable in terms of access and quality [1].

MCDA: MCDA is a decision-making process that balances competing priorities and takes multiple criteria into consideration during evaluation. For instance, in healthcare, MCDA is used to determine how to allocate investments in areas including clinical effectiveness, cost, equity, feasibility, and stakeholder preferences [1]. Such a method ensures that complicated decisions are made in an even-handed fashion, with the many faces and oftentimes conflicting needs of healthcare systems taken into consideration.

MCDA is especially valuable for assessing large-scale projects, like infrastructure upgrades or new technologies, that create decisions regarding short-term and long-term impacts. For example, hospitals in underserved communities use MCDA to determine whether to invest in new surgical centers or improve emergency room capacity, weighing factors such as demographic needs, workforce availability, and expected patient outcomes [4]. Example applications: Hospitals can use MCDA to evaluate different technology solutions against metrics such as implementation costs, clinical outcomes, and integration with current systems. MCDA can help in this regard as it brings an evidence-based approach to ranking investment alternatives and determining the most advantageous solutions by weighting each related criterion [4].

MCDA is particularly strong because it can engage multiple stakeholders to ensure that investments incorporate a variety of views and priorities. Hospitals should integrate predictive analytics to strengthen MCDA’s effectiveness in resource planning. This approach encourages consensus and trust among all stakeholders, which is widely seen as a prerequisite for implementing system-wide improvement in the healthcare setting.

Cost-benefit and risk assessment models: Below are resources and models that offer cost-benefit analysis (CBA) and risk assessment methods for capital investments in healthcare. These models aim to assist hospitals in determining whether proposed investments are financially sound based on a comparison of costs to anticipated benefits, such as increased patient outcomes, operational efficiencies, and revenue [33,34].

For instance, hospitals investing in robotic-assisted surgery conduct CBAs to determine whether the high upfront costs are justified by improved surgical precision, reduced complications, and shorter hospital stays.

Thus, a common approach to this context is to use capital budgeting techniques, such as net present value (NPV) and/or internal rate of return (IRR), to assess the long-term financial returns of investment projects. NPV is a calculation of how much a given investment can net by taking the value of cash flows in the future and discounting it to the present, while IRR is an estimate of the rate of return someone can expect to make over the investment’s life. These methods guarantee that decisions are cost-effective and fit organizational financial objectives.

Other tools that can assist in making investments are risk assessment tools, which quantify the sensitivity analysis, and simulation models, which evaluate the risks and uncertainties inherent in an investment. Sensitivity analysis determines which key variables - like implementation costs or patient demand - most affect investment outcomes, and simulation models allow hospitals to explore multiple scenarios and identify optimal strategies [34].

These tools do just that, helping decide where capital is allocated to projects that provide measurable value, which aligns with strategic goals, and that improve long-term sustainability. Additionally, sensitivity analysis can evaluate the financial risks associated with variable patient demand, reimbursement changes, and technological obsolescence.

Strategies for Optimal Working Resource Allocation

Proper resource allocation is essential in guaranteeing that constrained healthcare resources are utilized effectively, allowing for maximum patient outcomes and operational efficiency. Hospitals use different strategies to ensure they balance resource shortages, ethics, and economic imperatives. Key strategies are optimization and predictive modeling, ethical and economic trade-offs, transparent prioritization frameworks, and disinvestment strategies. These methods enable healthcare organizations to make informed, systematic, and evidence-driven decisions [1,2].

Optimization and predictive modeling: They can utilize optimization and prediction methods to allocate resources, such as predicting the demand resource allocation while managing capacity and clinical workflow in an efficient manner. Predictive analytics use past and current data to enable forecasting of patient admissions, treatment, and staffing needs. Hospitals can enhance patient experience and improve service delivery through predictive insights to anticipate patient inflow trends and proactively allocate resources accordingly to avoid congestion and reduce wait times [35].

Additionally, hospitals integrating predictive modeling must ensure the ethical use of patient data and maintain transparency in AI-driven decision-making to foster stakeholder trust and regulatory compliance [36].

Simulation-based optimization models further optimize resource allocation to improve outpatient appointment scheduling and capacity planning. Heuristic algorithms can, therefore, be used to reduce patient wait times and the number of clinical staff working overtime alongside the efficient use of available resources [37]. Eliminating bottlenecks in healthcare delivery by using these tools allows for a higher level of operational efficiency while also increasing patient satisfaction by optimizing where resources can be applied.

Predictive tools can help with resource planning for peak-demand times, such as during pandemics or seasonal spikes. Hospitals can dynamically assign staff, equipment, and facilities to the multiple demand scenarios they simulate, which can also minimize resource underutilization or shortages [1]. Such data, in turn, can help ensure not just optimizing hospital operations but also preparedness for unpredictable healthcare demands.

Despite the benefits, predictive modeling requires continuous refinement, as inaccurate forecasting could lead to resource misallocation or unintended consequences in patient care [38].

Ethical and economic trade-offs: Ethical decision-making in resource allocation must also consider cultural and regional factors. What is deemed equitable in one healthcare system may not align with the priorities of another [39]. Resource allocation in healthcare often involves balancing ethical principles with economic constraints. There are two main approaches, utilitarian and egalitarian, to how to make decisions in this regard. Utilitarian aims seek to maximize the total health of the population and often allocate resources based on which programs or services will have the most significant impact on the largest number of people. This focus on cost-effectiveness and efficiency means that high-impact interventions and technologies are often favored [40].

Egalitarian goals, in contrast, aim to decrease health inequities and allocate care fairly across patients. While the best gain from the overall perspective would be through targeted health interventions, this approach addresses those on the margins or low services, including patients with HIV or multiple chronic illnesses [33,40]. Achieving a balance between these aims necessitates a nuanced understanding of the clinical and economic implications of both approaches.

Cost-effectiveness analyses are routinely utilized to measure trade-offs between utilitarian and egalitarian priorities. For instance, screening and/or treatment of the underserved may have lower cost-effectiveness in the short term than acute care interventions yet can transmit significant long-term health improvements. Evidence of fair and responsible allocation of resources to stakeholders results in transparent decision-making, which is in line with equity as well as efficiency objectives [2].

Additionally, ethical guidelines should be integrated into hospital governance policies to ensure that economic constraints do not disproportionately disadvantage vulnerable patient populations [41].

Publicly available prioritization frameworks: Transparent prioritization frameworks are vital to ensuring fairness, accountability, and stakeholder trust in resource allocation decisions. Stakeholder engagement - comprising clinicians, administrators, policymakers, and representatives of patients - has a dual role in identifying priorities as well as opportunity costs of alternatives and reaching a consensus regarding allocation decisions [1]. Engaging all relevant stakeholders ensures resource allocation accounts for multiple perspectives, clinical priorities, and organizational goals.

Frameworks must be adaptable to specific institutional needs, as rigid prioritization structures may fail to capture the dynamic nature of hospital operations [42].

Additionally, frameworks like PBMA and HTA can help ensure transparency by establishing systematic approaches to evaluating the costs and benefits of competing programs [1,2]. For example, PBMA outlined how to assess the marginal change obtained from the reallocation of funding from low-impact to high-impact programs, while the HTA process is nested in evaluating the clinical and economic value of new treatments.

From demand to supply, hospitals can make better decisions based on data by explicitly recognizing what is to be sacrificed to achieve the investment in question. When processes are transparent, it increases the confidence of stakeholders because allocation decisions are based on data, ethical principles, and strategic priorities [1].

Moreover, real-world applications of prioritization frameworks show that effective implementation depends on stakeholder engagement and institutional culture rather than just structured methodologies [43].

Disinvestment strategies: Disinvestment is very similar to the idea of divestment: you invest (disinvestment strategies like the disinvestment tool) in programs or services that provide less value so that you can have the resources to fund higher-impact initiatives. Disinvestment is a critical yet underutilized service and asset disinvestment component, as this dimension involves the disruption of organizational inertia and accounts for the opposition by stakeholders who might be invested in the status quo [1,2].

Disinvestment is a process that involves the assessment of healthcare services on the basis of their clinical effectiveness, cost-effectiveness, and fit with strategic objectives. Disinvestment decisions should be supported by empirical data to counteract resistance from stakeholders who may perceive such changes as cost-cutting rather than quality improvement [44].

Programs that struggle to illustrate improvements or efficiencies but for aggregate outcomes may be subject to reallocation, for example. The shift from failing services to effective services helps hospitals attract a better quality of service to their customers [2] and, therefore, provide even better value by implementing advanced technologies or specific clinical.

However, disinvestment also has potential challenges, including opposition from staff, patients, and stakeholders who view the cessation of programs as a loss of care. These challenges may be addressed by hospitals with transparent evaluation processes - based on evidence and stakeholder engagement - to justify disinvestment decisions. HTA and cost-benefit analyses are tools that can provide an objective framework for assessing program performance and help make this process easier [1].

Moreover, disinvestment should be seen as an ongoing process that evolves alongside changing healthcare priorities and evidence. Hospitals implementing disinvestment strategies must also develop communication plans to ensure transparency and minimize resistance from affected stakeholders [24].

Where to apply investment frameworks at clinical programs: Investment frameworks are critical for guiding resource allocation across infrastructure, technology adoption, specialized clinical programs, and crisis response. Hospitals can help to achieve this by leveraging evidence-based strategies and prioritization tools to ensure that their investments do so in a manner that is aligned with organizational goals and patient needs [1,5].

Expertise in managing infrastructure and facilities

The delivery of high-quality care depends upon infrastructure investments and facilities management (FM) to ensure operational efficiency. Facility upgrades in hospitals are typically funded through performance-based financing models that link investments to improved healthcare delivery and health outcomes [45]. FM, specifically the maintenance and optimization of physical infrastructure, directly affects patient satisfaction and operational performance.

The research evidence indicates that FM underfunding can result in inefficiencies and lower quality of care. Long-term sustainability is improved through strategic investments in infrastructure, e.g., operating room modernization, outpatient facility expansion, and energy-efficient systems [45]. Performance-based financing helps to ensure that hospitals are focused on targeted upgrades that provide maximal value, making sure that resource use is aligned with organizational priorities.

Additionally, investment in resilient infrastructure should consider climate adaptation strategies, as extreme weather events can disrupt hospital operations and compromise patient care. Hospitals must also balance short-term operational needs with long-term sustainability goals, ensuring that facility upgrades do not compromise financial flexibility [46].

Adoption of Healthcare Technologies

A critical application of investment frameworks is the adoption of H4.0 technologies such as telemedicine platforms, EHRs, and AI. In different ways, these technologies are enhancing patient care, optimizing workflows, and lowering operational costs [14].

A major challenge in adopting H4.0 technologies is the digital divide. Hospitals in lower-resource settings may struggle with the cost of implementation, interoperability with existing systems, and the training of healthcare personnel [47].

Frameworks like HTA enable hospitals to assess the clinical efficacy and cost-effectiveness of technologies prior to their rollout. Telemedicine, for example, played a critical role in ensuring the continued delivery of care and minimizing the risk of physical contact during the COVID-19 pandemic [33]. EHR systems improve care coordination, enable data accessibility, and guide evidence-based clinical decisions.

Importance of a holistic approach to technological investments: Hospitals need to take a purposeful view of technology investments in terms of their technological interdependencies, integration challenges, and stakeholder involvement to succeed [14]. For successful integration, technology investments must align with institutional capacity, stakeholder readiness, and clear return-on-investment projections to ensure long-term viability [14,48].

Specialized Clinical Programs

Such investment frameworks are important for prioritizing specialized clinical programs like mental health, geriatrics, and pediatrics. Despite their crucial impact on public health and quality of life, specialized clinical programs often suffer from chronic underfunding [49]. For example, mental health programs are critical in order to meet increasing mental health needs, especially in underserved populations [4].

For instance, investments in geriatric care solve the growing demand caused by aging populaces, ensuring hospitals can provide holistic care for older adults. Services provided by pediatric programs, including preventive services and acute care, are essential to improving life course health outcomes and lowering lifetime healthcare costs.

Investment decisions should also account for social determinants of health, ensuring that underserved populations receive equitable access to mental health, geriatrics, and pediatric services [50].

Frameworks such as PBMA assist hospitals in assessing and prioritizing investments in these specialized programs, enabling resources to be allocated toward areas of maximum marginal benefit [2].

Hospitals implementing PBMA for specialized programs should also establish clear outcome metrics to assess the long-term impact of investments on patient well-being and healthcare efficiency [51].

Crisis Situational Resource Allocation

Future crisis preparedness strategies should integrate flexible resource allocation models that allow hospitals to scale services rapidly without long-term financial strain. The COVID-19 pandemic highlighted the need for the strategic allocation of resources during times of healthcare crisis. Moreover, hospitals suddenly had to make investments in vital areas - ICUs, PPE, and digital health, among other needs - overnight to meet the demand surge and ensure continued delivery of care.

The crisis highlighted the need to have investment frameworks that include predictive analytics and risk assessment tools. Hospitals used telemedicine, for instance, to provide outpatient services while limiting in-person meetings [14,33]. Further, emergency resource allocation lends itself to adaptability, enabling hospitals to redeploy personnel and resources as needed in real time.

The pandemic underscored the importance of assiduously pursuing robust, evidence-based frameworks for consideration of crisis preparedness and systems to make timely, strategic investments during emergencies. Through data-driven methods, hospitals can reduce risks, improve resource usage, and build resilience to future adversities.

Beyond pandemic response, emergency investment frameworks should extend to disaster preparedness for climate-related events, mass casualty incidents, and cyberattacks, ensuring hospitals can maintain operations in diverse crisis scenarios [52].

Challenges in investment decision-making

Hospitals make investment decisions that are complex and involve financial, technological, and organizational perspectives. Although structured frameworks already exist, healthcare organizations are confronted with high-impact multi-factor drivers that prevent optimal resource allocation. This implies that decision-making is often ad hoc, with financial constraints, integration of new technologies, and discrepancies between stakeholder and policy needs [1,14]. There is a need to address these issues to promote evidence-based, sustainable, and equitable clinical program investments.

Historical and Ad Hoc Allocation Practices

Many health systems utilize historical allocation patterns and ad hoc decision-making processes, not systematic, evidence-based frameworks. Moreover, traditional approaches often allocate resources based on previous budgeting periods or on institutional inertia, which can prevent project efficiency and lead to poor investment outcomes [1]. Such patterns make it difficult to meet emerging healthcare needs, technological advances, or our patients’ ever-changing needs.

One major issue with historical allocation is the lack of a structured review mechanism to reassess whether past investment decisions remain relevant in the face of evolving healthcare needs and innovations [53].

For example, legacy programs may receive funding because that is what has been done historically, while more innovative or impactful programs may not get funding. Without transparent evaluation frameworks in place, decision-making becomes not only inconsistent but also unaccountable. To inform investments, hospitals can benefit from structured tools like HTA and PBMA to optimize change management investments cumulatively against measurable outcomes and strategic alignment [2]. Additionally, introducing performance-based funding models can help shift resource allocation toward data-driven, outcome-based investments rather than continuing funding based on tradition or institutional inertia [54].

Economic Pressures and Budget Constraints

Hospitals function within increasingly tight financial environments, and margins are shrinking; higher operating costs make a case for the allocation of capital even more challenging. Financial sustainability pressures are exacerbated by unpredictable reimbursement models and shifts in healthcare policy, making it difficult for hospitals to plan long-term capital investments. Small, rural, or independent hospitals experience strong financial pressures because they have limited access to external funding and face higher agency costs [7,8]. The financial strains lead health systems to focus more on short-term financial solvency than on investments in long-term facility infrastructure, technology, and clinical program expansion.

For-profit hospitals are likely to invest in services that generate immediate returns, such as revenue-intense services; not-for-profits provide community-oriented services with limited financial resources [20]. The challenge is how to deliver equitable, high-quality care while maintaining financial sustainability. Increasing financial analysis by employing cost-benefit analyses and risk assessment tools like NPV and sensitivity analysis will allow hospitals to make rational spending decisions despite cost limitations [34]. Collaborative financing approaches, such as public-private partnerships, could offer alternative funding solutions for hospitals struggling with capital constraints [55].

Incorporation of Technological Innovations

H4. 0 technologies - telemedicine, EHRs, and AI - have a great deal of potential to improve care delivery. One of the major barriers to successful technological adoption is the lack of standardized interoperability frameworks, which prevents seamless integration across different healthcare systems [47]. Nevertheless, hospitals do suffer from technological fragmentation, low interoperability, and high asset specificity [14,17]. Systematic interdependencies of individual technology investments can result in systems being out of sync, leading to limited overall system efficacy.

This challenge calls for in-depth methodologies for holistic evaluations, e.g., HTA, which studies the interrelation of technologies and long-term costs and needs of stakeholders [14]. Moreover, encouraging collaboration with stakeholders and enabling adequate training is imperative to ensure the successful implementation and integration of new technologies.

To mitigate these risks, hospitals should consider phased implementation strategies that allow for gradual integration while minimizing financial and operational disruptions [56].

Challenges From Stakeholders and Policies

Engagement of various stakeholders - such as different internal and external stakeholders like hospital administrators, clinicians, and policymakers - without alignment with regulatory requirements breeds substantial gaps in investment decision-making. Navigating complex regulatory environments, as well as managing the often conflicting priorities of multiple stakeholders [4]. Stakeholder misalignment often stems from differing risk tolerances between financial decision-makers and clinical leadership, leading to stalled investment approvals [57]. Regulatory compliance may, for example, demand substantial financial resources, taking money away from other highly impactful clinical programs.

The process is further complicated when disinvestment or program changes are proposed, as stakeholder resistance is common. For example, clinicians, patients, and community representatives might lobby to keep existing services despite being underperforming or out of step with organizational priorities [2]. Transparent prioritization frameworks and stakeholder engagement processes are critical to addressing these issues, building consensus, and guaranteeing that investment decisions align with institutional goals and regulatory obligations [1]. Hospitals should establish multi-stakeholder governance committees to improve investment alignment and ensure regulatory compliance while balancing financial and clinical priorities [58].

Future directions

As healthcare systems continue to evolve, so too must our investment decision-making to stay ahead of emerging challenges and opportunities. Future directions focus on strengthening transparency, utilizing advanced analytics, adopting new technologies, involving stakeholders, and ensuring sustainability. Such strategies will develop decision-making frameworks, optimize resource allocation, and determine long-term healthcare goals [1,14].

Next-Level Decision-Making Frameworks

However, for future investment decision-making, it is crucial to enhance transparency, equity, and formalized processes. Future decision-making frameworks must also integrate real-time data collection and adaptive learning mechanisms, ensuring that investment strategies remain responsive to evolving healthcare demands [36]. Ad hoc approaches need to be supplanted by structured frameworks that integrate clinical, financial, and ethical considerations, such as HTA and PBMA [1,2]. These frameworks will be utilities that ensure the maximization of equity in investment pipelines while aligning initiatives to institutional goals and patient needs with innovation.

Multi-criteria evaluations will be formally integrated into processes, and consideration will be given to opportunity costs and potential impacts. This also supports better decision-making accountability and trust among stakeholders in the process at scale to enable a more effective rollout of impactful investments. These frameworks could help hospitals refine their processes while addressing both their strategic goals for balancing resource allocation and equity in healthcare outcomes [1]. Additionally, decision frameworks must be designed with interoperability in mind, ensuring that hospitals can collaborate across different healthcare networks and data-sharing infrastructures [59].

Utilizing predictive analytics and AI: Predictive analytics and AI are well positioned to transform hospital investment decision-making through data-driven optimization of resources. AI, in particular machine learning, is able to analyze large volumes of historical and real-time data and is capable of predicting patient demand, treatment requirements, and operational bottlenecks. Such knowledge would enable hospitals to flexibly distribute resources as needed and preemptively solve potential challenges [35,37].

Beyond patient demand forecasting, AI applications in hospital investments extend to optimizing procurement strategies, managing supply chains, and reducing operational waste through automated resource allocation [60]. AI-powered investment appraisal tools simulate various economic and clinical scenarios, allowing hospitals to predict and mitigate financial risks before committing to large-scale investments [61]. Likewise, hospitals may track the use of AI to approximate the long-term effects of investing in new clinical pathways or technology, which must be monitored over time to confirm that relevant investments align with financial sustainability and patient outcomes. Predictive analytics can provide healthcare systems with the ability to identify trends, identify upcoming demands, and improve cost efficiency [1].

Harnessing H4.0 technologies: Telehealth, EHRs, and other H4.0 technologies necessitate specific evaluation frameworks that can adequately measure the interrelated value of new cross-sector digital components. H4. 0 technologies, which are interdependent and often require system-level adoption to reach their full potential [14].

There is a need for hospitals to create holistic evaluation frameworks that consider benefits over a longer time frame, substitutions with other technologies, interoperability, and scalability. One major challenge in adopting H4.0 is ensuring seamless integration with legacy systems, requiring significant financial and logistical planning to prevent workflow disruptions. Assessing bundled technology investments - for instance, AI tools integrated with EHR systems - can help ensure that decisions maximize efficiency and clinical outcomes. Assessment features will be customized to ensure the technological uncertainty is well managed while limiting the associated risks, allowing for assurance that returned value and investment are realized across the healthcare systems [14,17]. To address these challenges, investment in cross-platform interoperability solutions and standardized data protocols will be critical for long-term success [62].

Recognition of stakeholder-centric approaches: Engaging a variety of stakeholders in decision-making is key to ensuring that investments are responsive to the needs of patients, clinicians, administrators, or policymakers. Collaborative approaches promote consensus, enhance transparency, and develop ownership of resource allocation [1,4].

Structured frameworks that involve multiple perspectives and criteria in their evaluation process can also help incorporate stakeholders into the evaluation effort. Stakeholder engagement must go beyond consultation to co-decision-making, ensuring that investments reflect the collective priorities of the healthcare ecosystem rather than being dictated solely by financial or administrative bodies [63]. Decision-making frameworks such as MCDA empower stakeholders to compare priorities and ensure investments are targeted to clinical efficacy, fairness, and operational objectives [1]. Involving stakeholders early often improves the quality of decision-making and helps ensure that investment initiatives are well conducted and executed. Institutions should also consider implementing digital stakeholder engagement platforms, allowing real-time input from diverse groups, including patients, healthcare workers, and policymakers [64].

Sustainability and long-term value creation: Investment alignment with environmental, social, and long-term healthcare goals is a rising priority deemed necessary for hospitals to move forward. Sustainability-focused investment strategies should also incorporate circular economy principles, reducing medical waste and increasing the lifespan of healthcare assets [65]. Investments are sustainable: new infrastructure, energy-efficient systems, and health equity and community-focused programs [45]. Hospitals need to introduce life-cycle cost analyses to assess the long-term value of investments over short-term financial pressures.

Investment in sustainable infrastructure, for instance, lowers operational costs while enhancing environmental performance. This is also the case with those efforts that focus on underserved populations or chronic disease management, which offer the opportunity for improved long-term public health [4]. Hospitals can also drive their financial strength, social accountability, and value in patient care by embedding sustainability into investment decisions.

Furthermore, transparent sustainability reporting mechanisms can help hospitals track progress on environmental, social, and governance goals, ensuring accountability and continuous improvement [66].

## Conclusions

Investing in clinical programs is a multifaceted process informed by financial health, market needs, technological developments, and organizational objectives, to name a few. Frameworks such as PBMA, HTA, and EBD help guide resource allocation, ensuring decisions are evidence based, ethically sound, and aligned with the broader interests of the health system. Tackling today’s challenges, be they piecemeal approaches to decision-making, struggling with limited resources, or technological disintegration, necessitates novel methods of documentation and transparency. With detailed frameworks and analytics, healthcare organizations can find the right balance between resource allocation, patient outcomes, and sustainability over time.
